# Hybrid-3D robotic suite in spine and trauma surgery - experiences in 210 patients

**DOI:** 10.1186/s13018-024-05044-9

**Published:** 2024-09-14

**Authors:** Dominik M. Haida, Peter Mohr, Sae-Yeon Won, Thorsten Möhlig, Mike Holl, Thorsten Enk, Marc Hanschen, Stefan Huber-Wagner

**Affiliations:** 1 Department of Trauma Surgery, Technical University of Munich, Klinikum rechts der Isar, Ismaninger Straße 22, 81675 Munich, Germany; 2 Department of Trauma Surgery, Diakonie-Klinikum Schwäbisch Hall, Diakoniestraße 10, 74523 Schwäbisch Hall, Germany; 3 Radiation Protection, Diakonie-Klinikum Schwäbisch Hall, Diakoniestraße 10, 74523 Schwäbisch Hall, Germany; 4 Department of Neurosurgery, Rostock University Medical Center, Schillingallee 35, 18057 Rostock, Germany; 5 Department of Neurosurgery, Diakonie-Klinikum Schwäbisch Hall, Diakoniestraße 10, 74523 Schwäbisch Hall, Germany

**Keywords:** Robotics, Navigation, Robotic arm, Hybrid OR, Pelvis, Acetabulum, Spine surgery, Trauma surgery, Neurosurgery, Cone beam CT (CBCT)

## Abstract

**Background:**

In modern Hybrid ORs, the synergies of navigation and robotics are assumed to contribute to the optimisation of the treatment in trauma, orthopaedic and spine surgery. Despite promising evidence in the area of navigation and robotics, previous publications have not definitively proven the potential benefits. Therefore, the aim of this retrospective study was to evaluate the potential benefit and clinical outcome of patients treated in a fully equipped 3D-Navigation Hybrid OR.

**Methods:**

Prospective data was collected (March 2022- March 2024) after implementation of a fully equipped 3D-Navigation Hybrid OR (“Robotic Suite”) in the authors level 1 trauma centre. The OR includes a navigation unit, a cone beam CT (CBCT), a robotic arm and mixed reality glasses. Surgeries with different indications of the spine, the pelvis (pelvic ring and acetabulum) and the extremities were performed. Spinal and non-spinal screws were inserted. The collected data was analysed retrospectively. Pedicle screw accuracy was graded according to the Gertzbein and Robbins (GR) classification.

**Results:**

A total of *n* = 210 patients (118 m:92f) were treated in our 3D-Navigation Hybrid OR, with 1171 screws inserted. Among these patients, 23 patients (11.0%) arrived at the hospital via the trauma room with an average Injury Severity Score (ISS) of 25.7. There were 1035 (88.4%) spinal screws inserted at an accuracy rate of 98.7% (CI95%: 98.1-99.4%; 911 GR-A & 111 GR-B screws). The number of non-spinal screws were 136 (11.6%) with an accuracy rate of 99.3% (CI95%: 97.8-100.0%; 135 correctly placed screws). This resulted in an overall accuracy rate of 98.8% (CI95%: 98.2-99.4%). The robotic arm was used in 152 cases (72.4%), minimally invasive surgery (MIS) was performed in 139 cases (66.2%) and wound infection occurred in 4 cases (1,9%). Overall, no revisions were needed.

**Conclusion:**

By extending the scope of application, this study showed that interventions in a fully equipped 3D-Navigation Hybrid OR can be successfully performed not only on the spine, but also on the pelvis and extremities. In trauma, orthopaedics and spinal surgery, navigation and robotics can be used to perform operations with a high degree of precision, increased safety, reduced radiation exposure for the OR-team and a very low complication rate.

## Introduction

The modern era of surgery has greatly changed with the development and increasing use of navigation and recently robotics. Major developments have brought changes and improvements in a wide variety of fields, but are particularly noticeable in orthopaedic and spine surgery. The latter, due to its long history and great reputation, has always been at the cutting edge of progress. Therefore, it is not surprising that hybrid operating rooms (Hybrid ORs) are becoming more and more popular by increasing the possibilities (e.g., modern 3D imaging, immediate correction of implants or minimally invasive approaches) [1, 2]. Relevant descriptions of the treatment of spine and associated diseases with simple non-surgical and surgical methods have already been described approximately in the 5th to 4th centuries AD by none other than Hippocrates [3, 4].

With a few centuries in between, seen from our perspective, spine surgery reached almost today`s methodology. An example of this can be seen in the first conducted laminectomy by Cline in 1814 [5]. Besides laminectomies, there are nowadays more common procedures in spine surgery, such as spinal fusion and fixation, which were implemented later in time. For this type of interventions, the foundation was laid by Hadra in 1891. However, it took almost 60 years and many courageous researchers, before Boucher inserted the first pedicle screw back in 1959 [6]. Even today, pedicle screws are still one of the most advanced solutions for spinal stabilisation. As we have seen in the last two decades, progress is no longer focused on the fundamental type of technique; rather, the focus is more on improving quality of the current technique by increasing accuracy and safety. That is why it is important to continue developing and advancing navigation and, more recently, robotics.

The discovery of X-rays in 1895 by Wilhelm Conrad Röntgen, made imaging possible in the first place and thus created the basis for navigation [7]. While the basis remains the same, the equipment and techniques have been constantly developed further. Milestones have especially been made in the development of the C-arm (1955) and the computed tomography (CT) scanner (1972) [8]. The long period of use shows how groundbreaking these developments were and are. They have become an integral part of everyday practice. Further development now relates to various areas such as image quality, reduction of radiation exposure, or navigation. The first simple navigation methods were introduced at the beginning of the 20th century. Navigation has been able to develop in the direction of today`s standards from the end of the 1980s until today, thanks to these technical conditions and improved imaging. The application possibilities are diverse and not limited to a specific speciality, beginning mainly with the intracranial use (stereotaxis). Navigation has now spread to many areas such as the spine, pelvis, and extremities. Recently, development has taken another step forward, particularly for spine surgery, with the possibility of improved intraoperative imaging, which can be seen in the introduction of fluoroscopy, intraoperative computed tomography (iCT) and the development of 3D imaging with devices such as cone beam computed tomography (CBCT) [8]. Older devices have disadvantages such as a small field of imaging, low image quality and especially for iCTs, imaging must be carried out by specialised radiologic personnel [8, 9].

Today, the possibilities have improved and the application and handling have become better and simpler. Modern CBCT with scans performed by surgical assistance [9] have improved imaging quality. In addition, the large fields of imaging can be integrated very well into the operational workflow and enable the functional use of imaging-based robotics in so called Hybrid ORs.

In Hybrid ORs, the synergies between navigation and robotics can perfectly optimise treatment of the patient. Robotics, with its relatively short history in comparison to imaging and navigation, has so far mainly attracted attention through the use of the DaVinci (Intuitive Surgical, Freiburg, Germany) in medical specialities such as visceral surgery, urology and many others [10]. Recently, robotics has also been gaining ground in spine surgery, mainly with increasing use for the insertion of pedicle screws. Advantages are seen in the minimally invasive operation options with increased safety, greater precision and less trauma caused [11, 12].

Despite promising evidence in the area of navigation and robotics, publications describing the different systems have been lacking in case numbers and number of screws inserted and their focus has mainly been on just one area of intervention. For these reasons, the potential benefits previously mentioned have not yet been definitively proven [13–22]. Therefore, the aim of this retrospective study was to evaluate the potential benefit and clinical outcome of patients treated in a OR equipped with modern navigation systems and robotics solutions, i.e., a fully equipped 3D-Navigation Hybrid OR.

## Methods and material

A prospective data collection was started with the implementation of the 3D-Navigation Hybrid OR at the authors hospital (“Robotic Suite” consisting of a navigation unit “Curve Navigation System”, a robotic 3D cone beam CT, “Loop-X”, a robotic arm “Cirq Arm System”, a wall mounted monitor “BUZZ” and Elements Viewer 3D Mixed Reality; Brainlab, Munich, Germany). The author`s hospital is certified as a level I trauma centre (German Trauma Society - DGU) as well as a geriatric trauma centre (German Trauma Society).

All patients who underwent surgery since introduction of the 3D-Navigation Hybrid OR in March 2022 were included consecutively and prospectively until March 2024. The analysis was carried out retrospectively. Indications for surgery were trauma, tumor (oncology), spondylodiscitis, degenerative and rheumatic diseases. Interventions were performed in the regions of the cervical spine, thoracic spine, lumbar spine, pelvis (pelvic ring and acetabulum), extremities and maxillofacial.

Preoperative and postoperative CT scans were mandatory for inclusion in this study.

A positive ethics vote was issued by the responsible authority, the ethics committee of the Landesärztekammer Baden-Württemberg (Reference No. F-2024-037). The study was retrospectively registered in the German Clinical Trials Register on the 09/07/2024 (ID: DRKS00034551) (Images1–12).

**Image. 1:**
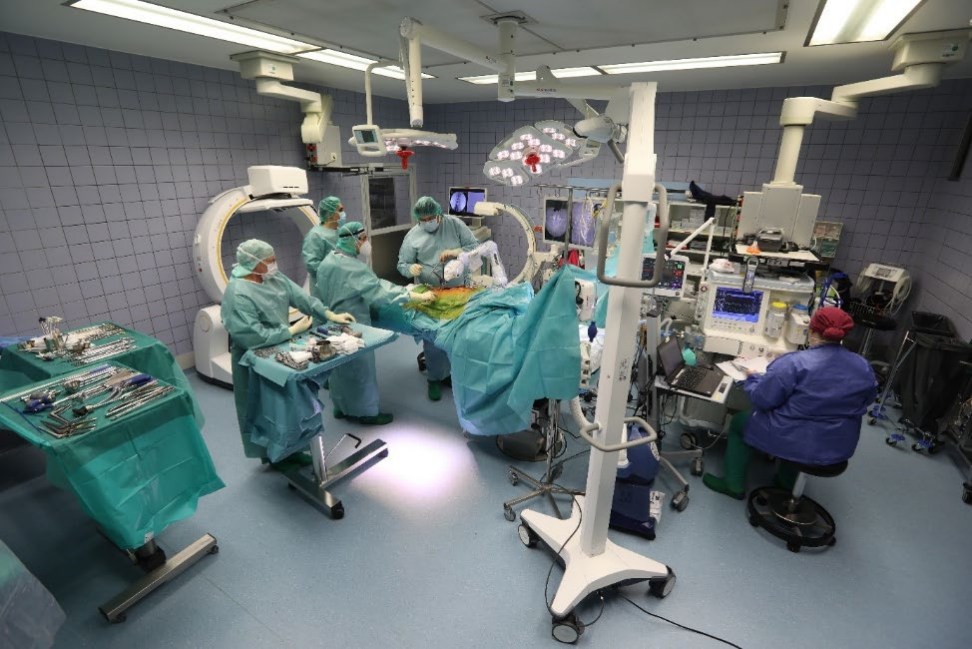
Overview of the 3D-Navigation Hybrid OR

**Image. 2:**
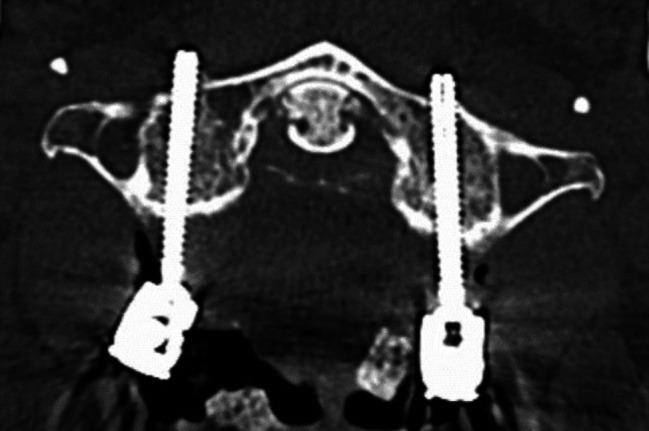
C1 lateral mass screws. *C*,* cervical spine*

**Image. 3:**
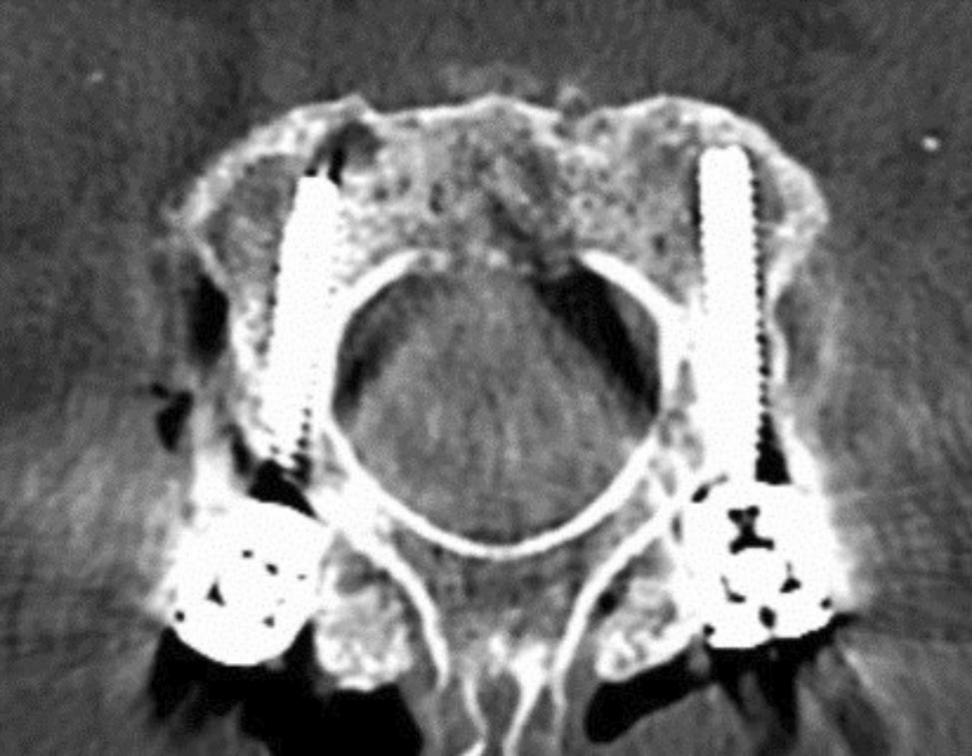
C2 isthmus screws. *C*,* cervical spine*

**Image. 4:**
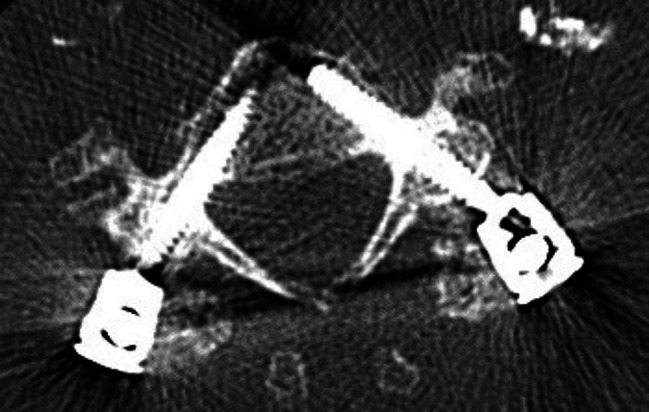
C3 pedicle screws. *C*,* cervical spine*

**Image. 5:**
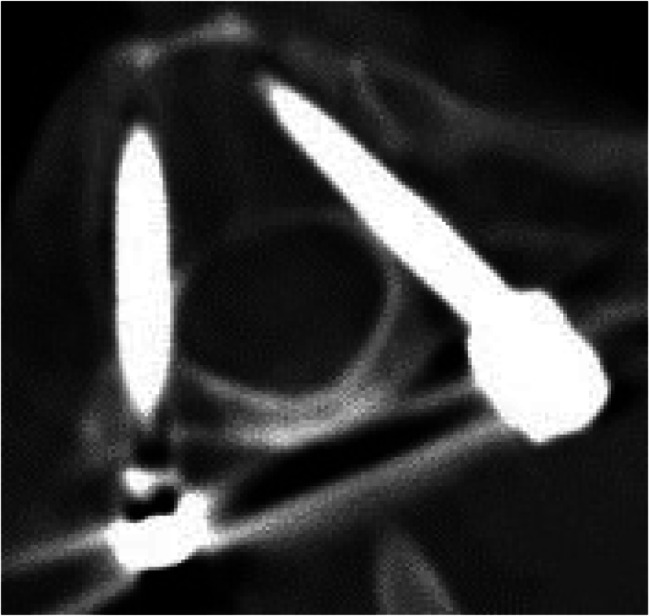
T1 pedicle screws. *T*,* thoracic spine*

**Image. 6:**
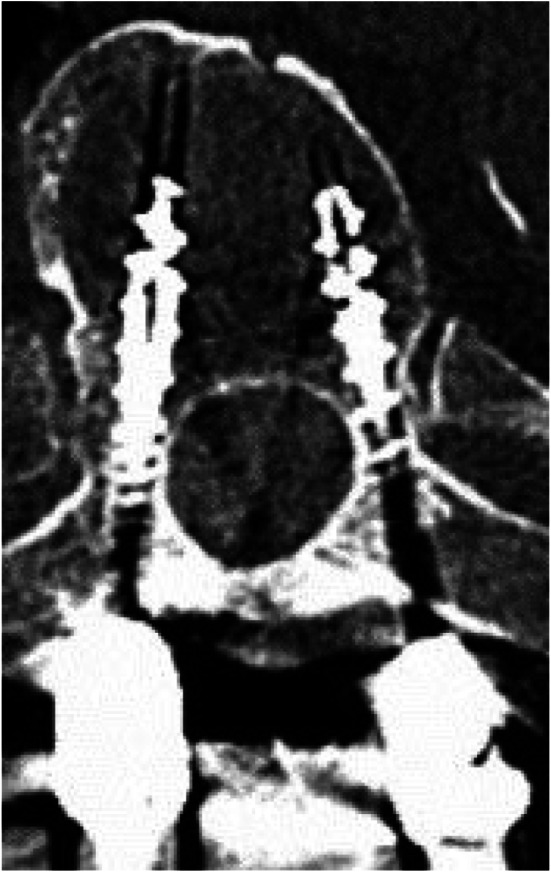
T7 pedicle screws. *T*,* thoracic spine*

**Image. 7:**
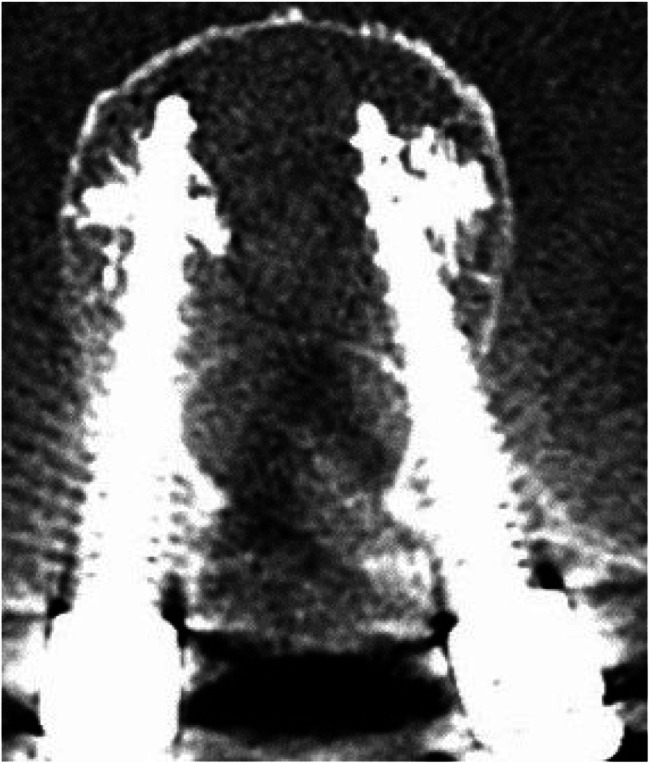
L1 pedicle screws. *L*,* lumbar spine*

**Image. 8:**
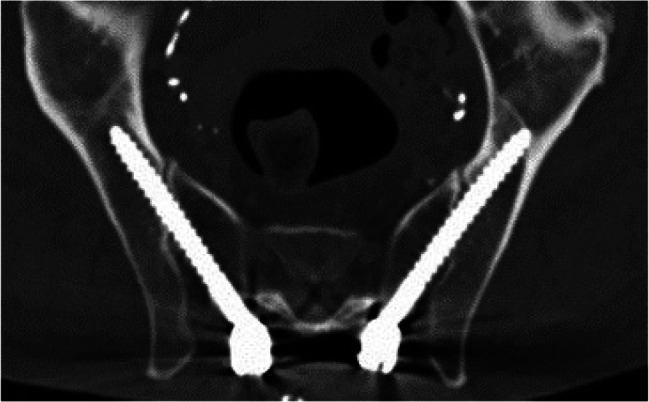
S2-alar-iliac screws axial (same patient as in image 9). *S*,* sacral spine*

**Image. 9:**
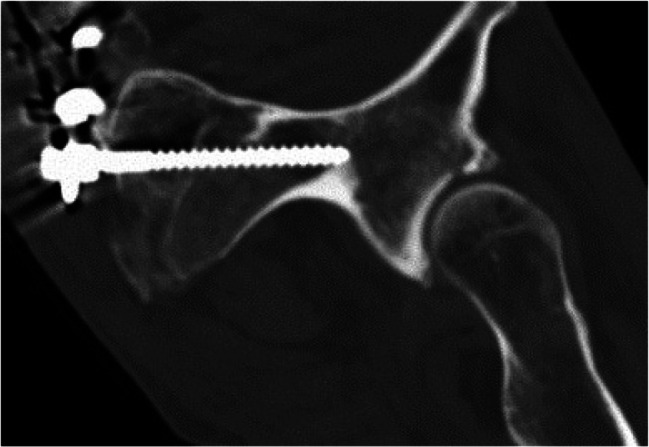
S2-alar-iliac screws sagittal (same patient as in image 8). *S*,* sacral spine*

**Image. 10:**
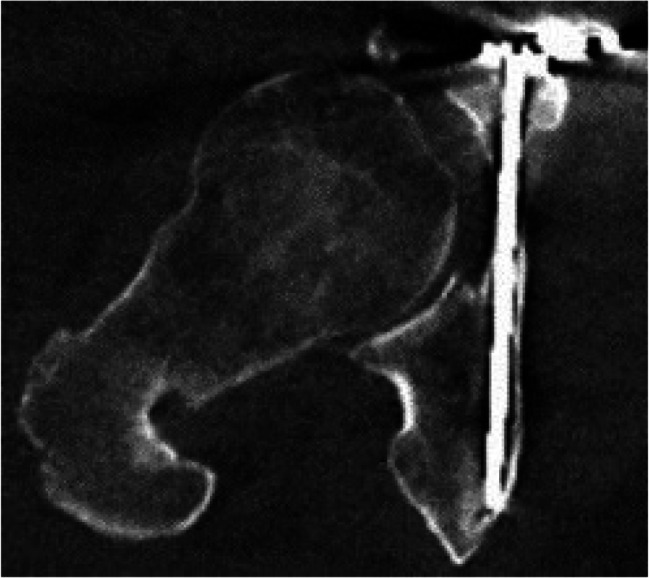
Infra acetabular posterior column screw rt. axial; posterior hemitransverse fracture according to Letournel (same patient as in image 11)

**Image. 11:**
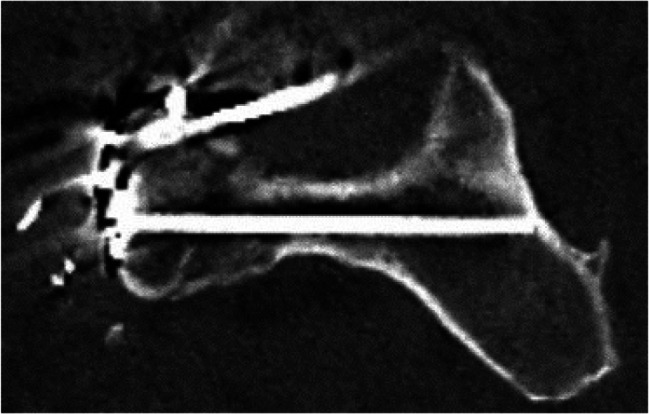
Infra acetabular posterior column screw rt. sagittal; posterior hemitransverse fracture according to Letournel (same patient as in image 10)

**Image. 12:**
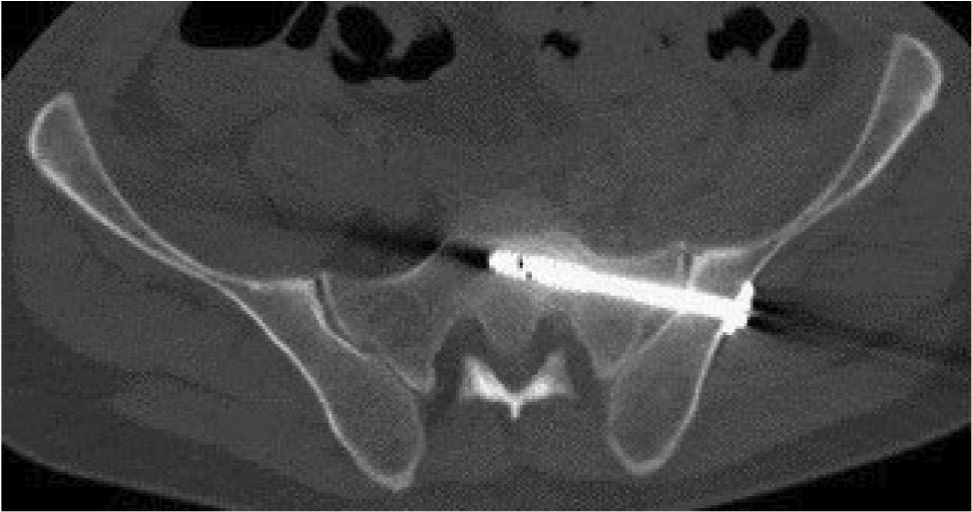
Sacroiliac (SI) screw S1 (same patient as in image 13). *S*,* sacral spine*

**Image. 13:**
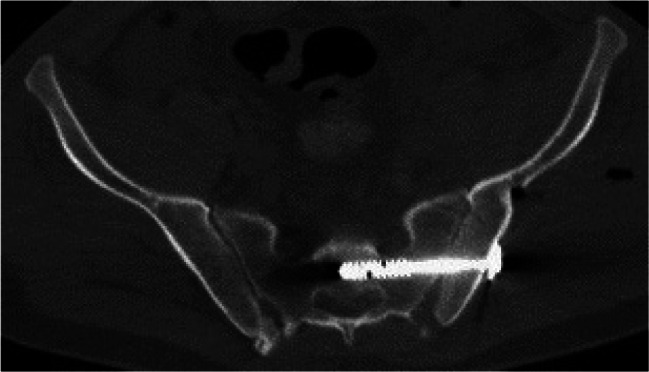
Sacroiliac (SI) screw S2 (same patient as in image 12). *S*,* sacral spine*

### Operative workflow

The steps of the workflow are described very well in existing literature. Here we refer in particular to the workflow described by Pojskić et al. [18], which is similar to ours.

The following is a step-by-step description of our workflow for spinal surgeries in our 3D-Navigation Hybrid OR.

#### Basic requirement

- Team Training

#### Preoperative

- Preoperative CT and/or Magnetic Resonance Imaging (MRI)
- Preoperative screw planning

#### Intraoperative

- Skin incision and placement of the reference star
- 1st “Loop-X” -scan (apnea; 100% oxygen)
- Data fusion of preoperative CT scan and 1st intraoperative CBCT scan (method: Automatic Image Registration “AIR”)
- Accuracy check with the pointer
- Referencing of the instruments
- In case of minimally invasive surgery (MIS) procedure: skin incision planning with the robotic arm; performance of the surgical approach; drilling and insertion of the K-wires navigated by hand or robotically assisted
- In case of an open procedure: performance of the surgical approach; drilling and insertion of the K-Wires navigated by hand or robotically assisted
- 2nd “Loop-X” -scan (apnea; 100% oxygen) and position evaluation of the inserted K-wires
- Tapping (optional)
- Screw insertion and cement augmentation (optional)
- Decompression/Laminectomy (optional)
- Attachment of the rods
- Wound closure

#### Postoperative

- X-Ray and/or CT-Scan (optional)

Surgeries were mainly performed by three experienced main surgeons from the department of trauma and spine surgery. The surgeons had already had years of experience in the conventional techniques of the respective interventions as well as in conventional hand navigation techniques.

The instruments used for interventions in the region of the spine were Solera (Medtronic, Meerbusch, Germany) and Viper Prime (DePuy Synthes, Umkirch, Germany) for thoracic and lumbar interventions, Symphony (DePuy Synthes, Umkirch, Germany) for cervical interventions. For interventions with osteosynthesis on the pelvic ring and the extremities, predominantly instruments by Königsee Implantate (Allendorf, Germany) were used.

### Data acquisition

#### Basic and descriptive data

Basic descriptive data and the intervention data from the patients (e.g., age, gender, indication of the surgical intervention, anatomic region of the intervention and number of screws) were collected from the surgical report. The number of intraoperative blood transfusions (erythrocyte concentrates) was counted based on the anaesthesia protocol. For categories such as length of hospitalisation, time at the ICU, incision-to-suture time, mortality rate and infection rate (without spondylodiscitis), the acquisition was carried out with the operation documentation, the surgical report, clinical documentation, letter of release and with the help of the accounting management. Data from trauma room patients (e.g., Injury Severity Score (ISS)) were available from the clinical documentation (shock room protocols). Average incision-to-suture time, hospital length of stay and length of ICU stays were calculated once for all interventions, once for interventions with and once without laminectomies and decompressions, as these interventions are very individual and often cannot be carried out using a standardised procedure. This means that they are not comparable with other operations and would therefore affect the comparability of the data. Preoperative and postoperative CT-scans were used for the analysis of the screw positions. The categories of interest were defined prior the inception of the study.

#### Radiation data

The X-ray system “Loop-X” is mainly intended to acquire CBCT data (three-dimensional (3D) data) during surgery. In addition, “Loop-X” can be used in a two-dimensional (2D) mode for images and fluoroscopy. The default settings for the 3D CBCT scans are a peak voltage of 120 kV and a scanning angular range of about 180 degrees (i.e., data for a typical CBCT are collected in a half rotation). These default settings were used in most of the interventions. A lower voltage was used only in a few protocols for extremities (e.g., feet).

The 3D data from the CBCT scans and the 2D data were automatically transferred to the picture archiving and communication system (PACS) of the hospital (Infinitt, Frankfurt/Main, Germany). The data included a digital imaging and communications in medicine (DICOM) radiation dose structured report (RDSR) object with a detailed table of all X-ray irradiation events (2D and 3D). The RDSR along with the dose information was automatically forwarded to the dose management system (DoseM, Infinitt) for further processing. Thus, the dose information from the “Loop-X” was well accessible for the surgery under discussion in the present study. However, due to technical problems in the early stage of operation, the automatic data transfer failed in five cases. These five cases are included in the discussion of the surgery, but excluded in the analysis of the dose data provided in this study.

In addition to the 2D and 3D X-ray applications from “Loop-X”, further 2D X-ray fluoroscopy with a C-arm (Ziehm Imaging, Nuremberg, Germany) was used throughout the surgery (see the above discussion of the operative workflow). At the end, dose information was extracted manually either from the dose sheet of the C-arm or from the legend in the C-arm images.

Due to the combination of 2D (from “Loop-X” and C-arms) and 3D (from “Loop-X” only) data, the dose information is given as dose area product (DAP) in the following. The computed tomography dose index (CTDI) for the 3D data was not used because a summing between CTDI doses for the 3D part and DAP doses for the 2D part is not straightforward. There were two further arguments why the CTDI was not used in the present study. (i) The definition of the CTDI implicitly assumes that irradiation occurs from all directions (like in a standard CT scanner). The CTDI for our CBCT with the typical irradiation in a restricted angular range is thus not well-defined because of the huge differences in the peripheral contributions to the CTDI. Furthermore, the usage of a CTDI for CBCTs requires special care because of the differences in the beam geometries between a CBCT and a standard CT; see e.g. the discussion in Alaei et al. [23] and references therein. (ii) The RDSR from the “Loop-X” provides only DAP data, but no CTDI for the 3D irradiation events.

The DAP was presented separately for the 3D contribution (“Loop-X”), the 2D contribution (“Loop-X”), and the 2D contribution from the C-arm. The total DAP was calculated from the sum of all contributions.

The data collected from the patients was systematically transferred to a table for analysis with the corresponding functions used for overall evaluation and measurement.

### Data analysis

#### Measurement of screws accuracy

The accuracy of spinal pedicle screw position (C1-S1) was independently measured by two researchers (Huber-Wagner and Haida) and graded according to the Gertzbein and Robbins (GR) classification [20, 24]:

- **GR-A**: no breach of the pedicular cortex, complete intrapedicular screw.
- **GR-B**: < 2 mm breach;
- **GR-C**: 2–4 mm breach;
- **GR-D**: < 6 mm breach;
- **GR-E**: > 6 mm breach [20, 24].

For interventions where larger screw diameters than pedicular diameter were required, the screws were counted as GR-A, when they penetrated the pedicle centred in its exact geometry.

**GR-A** and **GR-B** were defined as an accurate surgical result; otherwise they were defined as suboptimal/inaccurate screw placement (GR-C, GR-D, GR-E). The overall accuracy was calculated by the number of GR-A and GR-B screws divided by the total number of inserted screws and the calculation of the 95% confidence interval (95% CI), where appropriate.

Non-spinal screws could not be evaluated according to a classification, as there are no classifications existing for these areas of intervention, such as the Gertzbein and Robbins classification [20, 24] for the spine. Therefore, non-spinal screws were compared to their initial planned position. If the screws corresponded to the position planned, it was defined as a clinically satisfactory screw placement (almost comparable to a GR-A or GR-B position); otherwise it was defined as an inaccurate screw placement.

## Results

### Basic characteristics with indication and areas of intervention

In this study, *n* = 210 patients underwent surgical treatment in the Hybrid OR. The treatments were carried out on 118 (56.2%) males and 92 (43.8%) females with an average age at time of intervention of 66.7 (± 17.3) years and an average BMI of 25.4 (± 6.3). Out of the 210 patients, 23 (11%) arrived via the hospital’s trauma room. Of them, 17 (8.1%) patients had an ISS > 16, the average ISS of all trauma room patients was 25.7 (± 17.3). Details are illustrated in Table 1.

### Descriptive data

**Table 1:** List of the basic demographic and descriptive data. *ISS*,* injury severity scale; BMI*,* body mass index*

Variable	Value
Number of patients	210
Gender: Number of Male : Female (%)	118 : 92 (56.2 : 44.8)
Average age at time of intervention in years (standard deviation (± SD))	66.7 (± 17.3)
Average BMI (± SD)	25.4 (± 6.3)
Number of trauma room patients (%)	23 (11.0%)
Number of trauma room patients with an ISS ≥ 16 (%)	17 (8.1%)
Average ISS of trauma room patients (± SD)	25.7 (± 17.3)
Number of in-hospital mortality (%)	13 (6.2%)
Number of infections (%)	4 (1.9%)

The distribution of the indications for surgical treatment were; 183 (87.1%) fractures, 81 (38.6%) osteoporosis, 44 (21.0%) tumor cases, 14 (7.0%) deformity cases, 8 (3.8%) degenerative diseases, 6 (2.9%) spondylodiscitis, 4 (1.9%) Morbus Bechterew, 2 (1.0%) rheumatic diseases and 2 (1.0%) patients presented with a preoperative dural leakage. 29 procedures (13.8%) were performed on the cervical spine, 89 (42.4%) on the thoracic spine, 79 (37.6%) on the lumbar spine, 57 (27.1%) on the pelvic ring, 13 (6.2%) on the extremities 10 (4.8%) on the acetabulum, and 2 (1.0%) maxillofacial. Details are illustrated in Table 2. Some indications and procedures overlapped, therefore some patients were allotted to multiple groups.

### Anatomic regions and indications

**Table 2:** Sum of all intervention areas and diagnosis/indications

Intervention areas (partially overlapping)	Number of patients (%)
Cervical spine	29 (13.8%)
Thoracic spine	89 (42.4%)
Lumbar Spine	79 (37.6%)
Pelvic ring	57 (27.1%)
Extremities	13 (6.2%)
Acetabulum	10 (4.8%)
Maxillofacial	2 (1.0%)
**Diagnosis/Indications (partially overlapping)**	
Fracture	183 (87.1%)
Osteoporosis	81 (38.6%)
Tumor	44 (21.0%)
Deformity	14 (7.0%)
Degenerative diseases	8 (3.8%)
Spondylodiscitis	6 (2.9%)
Morbus Bechterew	4 (1.9%)
Rheumatic diseases	2 (1.0%)
Preoperative dural leakage	2 (1.0%)

### Surgery and postoperative clinical course

A robotic arm was used in 152 (72.4%) cases. In 139 (66.2%) cases, MIS techniques were used partially or completely. Additionally, microsurgical techniques (microscope, exoscope or magnifying glasses) were used in 55 (26.2%) cases, predominantly for laminectomies and decompression procedures. While Symphony was used for the C-Spine, Solera was used for the T-Spine and L-Spine with the exception of three cases. In these three cases, the Viper Prime system was used. Details are illustrated in Table 3.

### Surgical facts

**Table 3:** Sum of data from the surgical interventions. MIS, minimal-invasive surgery; TLIF, transforaminal lumbar interbody fusion

Operative factors	Number of patients (%)
Robotic arm in use	152 (72.4%)
MIS	139 (66.2%)
Cement augmentation	104 (49.5%)
Microsurgical technique	55 (26.2%)
Laminectomies/decompressions	42 (20.0%)
Intraoperative blood transfusions (erythrocyte concentrate)	19 (9.0%)
Kyphoplasty	15 (7.1%)
TLIF	7 (3.3%)
Thermal ablation	7 (3.3%)
Preoperative embolization	5 (2.4%)
**Instruments used**	
Medtronic Solera	109 (51.9%)
DePuy Synthes Symphony	30 (14.3%)
DePuy Synthes Viper Prime	3 (1.4%)
Other systems	68 (32.4%)

The average incision-to-suture time for all interventions was 197 (± 112) minutes. To specify the results, the average incision-to-suture times were also calculated separately. The average incision-to-suture time for interventions without laminectomies and decompressions was 173 (± 79) minutes and 290 (± 82) minutes for interventions with laminectomies and decompressions. Details are illustrated in Table 4.

### Time Analysis

**Table 4:** Analysis of the operation times

Duration of operations	Time in minutes (± SD)
Average incision-to-suture time for all interventions	197 (± 112) Range (minimum (min.)/maximum(max.)): 35–553
Median incision-to-suture time for all interventions	187
Average incision-to-suture time for interventions without laminectomies and decompressions	173 (± 79) Range (min./max.): 35–456
Median incision-to-suture time for interventions without laminectomies and decompressions	167
Average incision-to-suture time for interventions with laminectomies and decompressions	290 (± 82) Range (min./max.): 133–557
Median incision-to-suture time for interventions with laminectomies and decompression	278

Intraoperatively, 19 (9.0%) patients received blood transfusions (erythrocyte concentrate).

According to the Centers for Disease Control and Prevention (CDC) classification for surgical site infections [25], an infection occurred postoperatively in 4 cases (1.9%). The in-hospital mortality rate was 6.2% (13).

The average hospital length of stay for all patients was 17.0 (± 11.4) days. For patients who underwent surgery without laminectomies and decompression, the average hospital length of stay was slightly shorter at 15.1 (± 8.5) days. For patients who underwent surgeries in which laminectomies and decompression procedures were additionally performed, the average hospital length of stay was 24.2 (± 17.1) days. Of 210 patients, 69 (32.9%) had a postoperative ICU stay with an average of 1.3 (± 3.8) days. Details are illustrated in Table 5.

### Hospitalisation data

**Table 5:** Details about the hospital length of stay and ICU stays

Hospital length of stay & ICU stays	Time in days (± SD) / Number of patients (%)
Average hospital length of stay for all interventions	17.0 (± 11.4)
Median hospital length of stay for all interventions	14.0
Average hospital length of stay for interventions without laminectomies and decompressions	15.1 (± 8.5)
Median hospital length of stay for interventions without laminectomies and decompression	13.0
Average hospital length of stay for interventions with laminectomies and decompressions	24.2 (± 17.1)
Median Hospital length of stay for interventions with laminectomies and decompressions	17.0
**ICU stays**	
Total number of patients with an ICU stay	69 (32.9%)
Average length of ICU stays for all interventions	1.3 (± 3.8)
Number of patients with an ICU stay for interventions without laminectomies and decompressions	47 (22.4%)
Average length of ICU stays for interventions without laminectomies and decompressions	1.2 (3.8)
Number of patients with an ICU stay for interventions with laminectomies and decompressions	22 (10.5%)
Average length of ICU stays for interventions with laminectomies and decompressions	1.9 (3.8)

### Radiation dose

The total radiation dose was separately analysed in patients with spinal or non-spinal surgical treatment. The reason for the separate analysis was that typical non-spinal interventions on the extremities require significantly lower dose compared to spinal interventions due to the lower diameter of the irradiated areas.

It is obvious that the DAP for the patient depends on the dose and on the irradiated area. Depending on the surgical requirements, the “Loop-X” offers 3D scans with a large or small field of view (FOV). Thus, the irradiated area is a result of the clinical situation. The required dose of the 3D scans increases with the absorption in the patient and is hence related to the effective thickness of the patient. For spinal surgery, the effective thickness of the patient scales approximately with the body mass index (BMI). The full dose information was available for analysis from 205 of the 210 cases included in this study.

The resulting DAP values are listed in Table 6. We provide the DAP per 3D scan, the total DAP over all 3D scans, the DAP from the additional 2D irradiations with “Loop-X”, the DAP from the C-arm, and the total DAP of the whole OP. All DAPs are given in units of cGy cm^2^ (Table 6).

### Radiation

**Table 6:** List of dose area products (DAPs) for the different indications and scans. DAPs are given in units of cGy cm^2^

Radiation at spinal interventions for 141 patients	Number (± SD) / DAP (± SD)
Average number of 3D-scans	2.12 (± 0.54)
Average dose area product per 3D scan	1016 (± 743)
Average dose area product of all 3D scans	2154 (± 1771)
Average dose area product for “Loop-X” 2D scans	128 (± 140)
Average dose area product C-arm scans	962 (± 1090)
Total average dose area product	3244 (± 2265)
**Radiation at non-spinal interventions for 64 patients**	
Average number of 3D-scans	1.81
Average dose area product per 3D scan	1101 (± 686)
Average dose area product of all 3D scans	1996 (± 1542)
Average dose area product “Loop-X” 2D scans	108 (± 95)
Average dose area product for C-arm scans	545 (± 676)
Total average dose area product	2649 (± 2016)

### Accuracy of screw placement

**Fig. 1:**
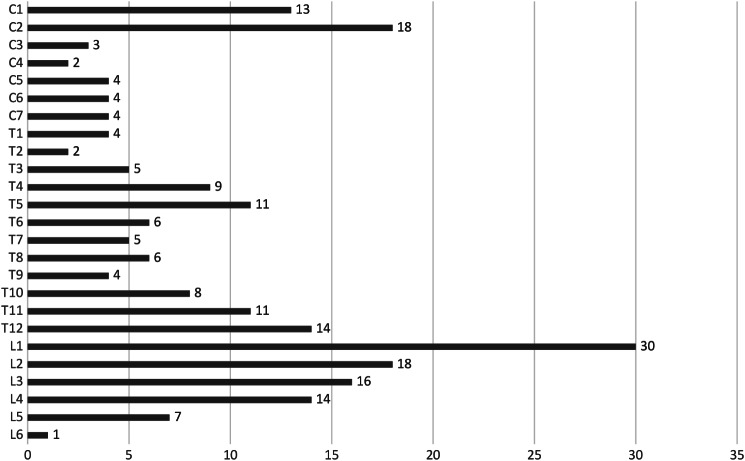
Injury per spine level. Ordinate shows the lesion heights; Abscissa shows the number of lesions at the respective height. *C*,* cervical spine; T*,* thoracic spine; L*,* lumbar spine*

The lesion heights of the spinal patients are summarized in Fig. 1.

#### Accuracy of spinal screw placement (spinal)

An average number of 5.6 (± 3.6) screws were inserted and 3.9 (± 1.5) segments were bridged in spinal patients.

In total, 1171 screws were implanted. Of them, 1035 spinal screws (88.4%) could be graded according to Gertzbein and Robbins [20, 24]. 911 screws were graded GR-A and 111 screws were graded GR-B. This resulted in an accurate screw placement of 98.7% (CI 95%: 98.1 − 99.4%). Although 1.3% of screws were inaccurately placed (GR-C: 7 screws, GR-D: 5 screws, GR-E: 1 screw), this did not cause any neurological deficits (Table 7).

#### Accuracy of pelvic and extremity screw placement (non-spinal)

A total of 136 (11.6%) non-spinal screws were inserted on the pelvic ring, the acetabulum and the extremities, of which 135 (99.3%) were correctly placed and 1 (0.7%) was suboptimally placed. Consequently, the rate of accurate placement of non-spinal screws was 99.3% (CI 95%: 97.8 − 100.0%) (Table 7).

#### Overall accuracy of screw placement

The overall accuracy for spinal and non-spinal screw insertion combined was 98.8% (CI 95%: 98.2 − 99.4%).

All in all, no revisions were required either in spinal or no-spinal screws resulting in 0% revision rate.

### Accuracy

**Table 7:** Analysis of the accuracy for spinal and non-spinal interventions

Variable	Number (%) / Number (± SD)
Total number of screws inserted	1171 (100%)
Spinal screws inserted	1035 (88.4%)
Non-spinal screws inserted	136 (11.6%)
Average number of inserted screws per patient	5.6 (± 3.6)
Average bridged segments (spine)	3.9 (± 1.5)
Revisions	0 (0%)
**Grading of spinal screws with the** Gertzbein and Robbins (GR) classification [20, 24]	
GR-A	911 (88.0%)
GR-B	111 (10.7%)
GR-C	7 (0.7%)
GR-D	5 (0.5%)
GR-E	1 (0.1%)
GR-C, GR-D, GR-E with neurological symptoms	0 (0.0%)
Suboptimally placed	13 (1.3%)
Accuracy spinal screw insertion: ((GR-A screws + GR-B screws) / Total spinal screws) * 100 = % of GR-A & GR-B	((911 + 111) / 103) *100 = 98.7% CI95%: 98.1 − 99.4%
**Grading of non-spinal screws**	
Correctly placed	135 (99.3%)
Suboptimally placed	1 (0.7%)
Accuracy non-spinal screw insertion: (Screws correctly placed / total non-spinal screws) * 100 = Accuracy	(135 / 136) * 100 = 99.3% CI 95%: 97.8 − 100.0%
**Overall malposition rate**	
Overall malposition rate: ((GR-C + GR-D + GR-E + suboptimally placed screws) / total screws) *100 = Total malposition rate	((7 + 5 + 1 + 1) / 1171) *100 = 1.2% CI 95%: 0.6 − 1.8%
**Overall accuracy**	
Overall accuracy: ((Gr-A screws + GR-B screws + correctly placed screws) / total screws) * 100 = Total accuracy	((911 + 111 + 135) / 1171) *100 = 98.8% CI 95%: 98.2 – 99.4%

## Discussion

To the best of our knowledge, this study is one of the largest studies reporting the outcome of navigated and robotic assisted surgery in spine, trauma and orthopaedics. 1171 screws were inserted into 210 patients at a high accuracy of 98.8%, a low complication rate and 0% revision rate.

### Operative workflow

The large number of operations with robotic assistance is mainly attributed to its practical applicability. Due to its attachment to the table, the robotic arm is easy to reach and does not restrict the surgeons’ mobility. However, the optimal preoperative fixation setup must be determined prior to the operation by the surgeon. The layout of the Hybrid OR with the attached robotic arm and two monitors, which ensure that several surgeons have a clear view even if they are on different sides of the operating table, improves ergonomics for the surgeons since less manual holding is required and everything is clearly visible to everyone from a comfortable position. It is also possible to work without an X-ray apron with a suitable workflow. All these factors are not unimportant considering the frequent musculoskeletal problems among surgeons that often arise in demanding fields such as spine surgery [26].

The presence of a technical employee from the manufacturer contributed to an optimal workflow during the operations. This is analogous to the field of heart and thoracic surgery, where a perfusionist is present when a heart-lung machine is in use. With one exception, there were no technical problems with the application. One unique problem occurred shortly after the implementation of the Hybrid OR during an operation with an earlier software version, which was corrected by immediately updating to a newer software version after this operation. This problem meant that navigation and robotics were not available until the software was updated. This particular operation could not be navigated or robotically assisted and instead was performed based on scans from our CBCT.

### MIS techniques and infections

Almost 2/3 of the surgeries in this study were performed partially or completely with MIS techniques. This high ratio of MIS treatments could only be achieved by implementing navigation in conjunction with a robotic arm. Navigated skin incisions made it possible to minimise trauma to the surgical site. We assume that the high ratio of MIS treatments contributed to the low infection rate observed.

It is important to emphasise that in our experience gentle soft tissue management during the surgical approach and during the process of navigation is of the utmost importance.

It should also be emphasised that some patients in this study underwent chemotherapy. The infection rates observed in our study were below the reported infection rates found in the literature on different techniques of dorsal instrumentation. The infection rate/revision rate due to surgical site infections, ranged from 5% up to 10% in these studies [27, 28]. In a case series comparable to ours analysing 125 cases and robotically assisted pedicle screw placements, a very low infection rate of 0.8% was achieved [29]. This emphasises our assumption that robotically assisted surgery may reduce infection rate.

These results indicate there could be potential for expansion of MIS techniques to the cervical spine, where currently open procedures are standard.

### Operation times

Currently, we cannot support the argument that either navigation and robotics reduce or increase operation time. This is methodically not possible based the data we analysed because we did not have a control group (non-hybrid, non-robotic).

We consider a reduction in the duration of the operation to be quite plausible, but very dependent on multiple factors that influence it. Simplified imaging using CBCT can and will probably often provide an advantage here, especially in areas that are difficult to visualise using conventional C-arm imaging (e.g. upper cervical spine or a degenerated spine). However, it is important to emphasise, that the imaging and workflow with a CBCT has a learning curve. As it is often the case, the surgeon’s experience and handling of the imaging is decisive for the duration of the operation.

Depending on the literature source, the operating times for robotically assisted procedures were shorter [30–32], longer [33] or comparable [34] to other techniques. We believe that operation time can be shorter in some cases and longer in other cases depending on the technique used. However, the results, especially in relation to screw placement accuracy, seem to be superior compared to a conventional setting. Considering the favourable outcomes of MIS techniques, which were carried out in large numbers in our study, potentially longer operating times are only of minor concern.

### Radiation

The discussion of radiation exposure is twofold because exposure of the patient and the surgeon (and further staff) have to be studied separately. In this study, the DAP per scan for spinal and non-spinal interventions are almost identical because the non-spinal interventions are mainly based on pelvic surgery, which is very similar to spine surgery. The interventions at extremities included in this investigation show lower DAP, but the number of patients was too low to have a major impact on the average DAP per scan for the non-spinal interventions.

It is obvious that the radiation exposure of the surgeon can be reduced by a major amount in a Hybrid OR. 3D scans contribute about two thirds to the overall irradiation for spinal and about three quarters for non-spinal surgeries. Because these 3D scans have only relatively short duration times, it is possible that the surgeon and further staff can move away from the patient or even leave the OR during irradiation. This was not possible in earlier times because of the ongoing fluoroscopy during the interventions. This means that the dose for the OR team is definitely reduced in our setting compared to a setting where only conventional 2D scans (C-Arm) are used.

The radiation exposure of the patient was provided as DAP in the present study. The DAP is a precise physics-based measure of the radiation exposure. Many other studies discuss only the applied fluoroscopy time, which does not provide sufficient information to constrain the radiation exposure of the patient. Mendelsohn et al. [35] converted all radiation exposures in CT-based navigation to the effective dose in mSv for comparison of the different 2D and 3D exposures. On the one hand, such a conversion to mSv provides a good and simple estimate for the risk of the exposure. On the other hand, the conversion factors from DAP to mSv (for 2D exposures) and dose-length product (DLP) to mSv (for 3D exposures) depend on the exposed body part. These conversion factors show significant uncertainties and have changed over time (e.g., see Table 3 of Elbakri et al. [36] or Table 5 of Compagnone et al. [37]). For this reason, we do not provide a detailed discussion of the effective dose in mSv in this work. It should however be noted that a reasonably averaged conversion factor for the spine surgeries (based on Compagnone et al. [37]) is slightly below 0.2 mSv per Gy cm^2^, leading to an average dose of about 2 mSv per 3D scan or about 6 mSv for an average spine surgery (including the 2D contributions from the “Loop-X” and the C-arm). This coarse estimate for the effective dose is close to the findings in Mendelsohn et al. [35]. For pelvic radiography, the conversion factor is slightly lower at 0.125 mSv per Gy cm^2^. A range between 0.10 and 0.15 mSv per Gy cm^2^, depending on the irradiation geometry, is given by Chen et al. [38]. This leads to effective doses of about 1.5 mSv per 3D scan in this case.

It is pointed out in Mendelsohn et al. [35] that a control group was not available for comparison with conventional surgery techniques due to missing dose information. Unfortunately, this general statement also holds true for the present study. Nevertheless, we have been able to investigate the radiation exposure for about twenty spine surgeries with conventional techniques directly before the robotic navigation became available in our hospital. The average DAP of 3805 (± 3132) cGy cm^2^ from these conventional surgeries is close to the present average DAP of 3244 (± 2265) cGy cm^2^. Hence, it can be concluded that patient exposure during OP is not increased in our Hybrid OR. Furthermore, the improved precision of the new technique reduces the number of subsequent surgeries, which avoids additional exposure and leads to further reduction of the overall exposure of the patient.

### Treatment of multiple injured patients

A relevant number of patients with multiple injuries were treated in our 3D-Navigation Hybrid OR. We consider interventions in a Hybrid OR, to be extremely demanding for these patients. It must be analysed in advance exactly how the intraoperative procedures fit in the spatial and technical conditions so that adaptions can be made and problems can be avoided regarding the positioning of the robotic arm on the table and the CBCT in the room. It could happen that only parts of the complete treatment can be carried out with the help of robotics and navigation. In the case of multiple injured patients, the sum or even the complexity of the injuries can result in a demanding surgical treatment. Whole-body CTs within Polytrauma management [39] are already available as standard of care for operation planning (the navigation used requires a minimal layer thickness of 2 millimetres). An additional intraoperative CT scan or normal high-resolution X-ray images might be needed to clearly visualise any severe anatomical positioning or complex injury patterns if needed. This means that optimum care for seriously injured patients can be guaranteed. Even if only a damage control surgery is deemed appropriate (mainly for certain extremity and pelvic injuries [40]), our Hybrid OR can be beneficial for precise positioning of an external fixator.

### Surgical challenges

The 3D Hybrid OR offers unique possibilities for challenging cases by offering options that might not have been possible in a different setting. One example is the placement of biomechanically more optimal pedicle screws instead of lateral mass screws [41] in the upper cervical spine, where safety really matters. Due to the risks in this demanding anatomic area [42], freehand and conventional technology often reaches their limits or certain interventions cannot be carried out at all. Navigation and robotics with the right workflow can enable the surgeon, guaranteeing the patient better treatment. In their meta-analysis from 2023, Zhou et al. [33] emphasised that robotic assisted procedures had far better accuracy than freehand techniques at pedicle screw placements on the very frequently occurring lesion height C1 (13 lesions in this study). Furthermore, good results were achieved in other challenging anatomical regions such as the cervical-thoracic junction, at L5/S1 and when inserting S2-alar-iliac screws. These results show that robotics and navigations may have contributed to the feasibility of challenging anatomical surgical sites.

Robotic assisted surgery has certain pitfalls and limitations (e.g., software issues or reachability of the intervention sites) [42, 43], which can lead to unsatisfactory results. Precisely for this reason, care must be taken when performing operations at a challenging surgical site so that the patient benefits from the modern technology.

### Accuracy of the screw placement

#### Accuracy of spinal screw placement (spinal)

There are few studies existing to date with a technical setup similar to ours (same robotic arm and navigation, different imaging), with various statements regarding the accuracy rate of the screw placement [18, 19, 21, 44–46]. Farah et al. [19] reported a very low accuracy rate on the cervical spine of 67% (specified by Wu et al.) [45] and an overall rate of acceptable screws of 85.7% (graded according to the classifications of Neo and Heary) in spite of a relatively low number of patients and screws [19]. This accuracy rate is much lower than the accuracy rate achieved in our study and the accuracy rates provided by Pojskić et al. [18] (70 screws inserted thoracolumbar; accuracy rate 94.25% GR-A & GR-B) and Gabrovsky et al. [21] (97 screws inserted thoracolumbar; accuracy rate 100% Gr-A & GR-B). The difference in accuracy rates may be due to a variety of circumstances. In these three studies, imaging was performed with iCT or C-Arm scanners. A modern CBCT with a voxel size of 0.25 mm was used in our study. The differences in accuracy could therefore lay in the different imaging quality, which results in a different data basis for navigation and the robotic arm. In addition, the registration method as well as the method and quality of the image fusion can have an influence on the accuracy of the screw placement. Further differences could lie in the workflow, which must be optimally adapted to the circumstances and types of intervention. We emphasise that our study exceeds the size of the other studies in terms of screws and areas of interventions. A highly regarded study by Devito et al. [47] showed already in 2010 that good results with robotically assisted pedicle screw placement could be achieved (3271 cases; 98% clinical acceptance rate). A comparison with more recent published studies such as Kanaly et al. [48] (326 screws; accuracy rate 97.5% GR-A & GR-B) and Vardiman et al. [49] (348 screws; accuracy rate 97.7% GR-A & GR-B) shows that our setup can keep up with the very high accuracies reported in similar studies with a different technical setup.

In a large meta-analysis from 2019, Perdomo-Pantoja et al. [50] analysed 51,161 pedicle screws and found accuracy rates ranging from 90.5% (robotically assisted) up to 95.5% (CT navigated). However, the number of included studies on robotically assisted procedures (7 studies) was far smaller than the group of studies investigating other procedures such as freehand techniques (20 studies), fluoroscopic techniques (56 studies) and CT navigated techniques (20 studies). The main reason for this is due to the fact that robotics were not very widespread at that time and seemingly inferior to pure navigation. In one of the largest recent meta-analyses (77,360 screws) from Naik et al. [51], the pedicle screws placed with the help of robotics performed significantly better than conventional and purely navigated techniques as demonstrated by a lower number of misplaced screws and higher accuracy (97.6% pooled accuracy rate for robotically assisted inserted pedicle screws).

#### Accuracy of pelvic and extremity screw placement (non-spinal)

While purely navigated procedures have also been used for some time in orthopaedics and trauma surgery, there is little literature [52–55] on orthopaedics and trauma surgery with navigation and robotics, apart from its use on the spine. Clinical application has so far focused on the insertion of SI-screws [52, 54, 56]. In our study, robotically assisted insertion was performed for anterior and posterior pelvic ring osteosynthesis, sacroiliac screws (SI-screws), acetabular screws as well as S2-alar-iliac-screw insertion. To the best of our knowledge, none respectively only a few clinical studies have reported on the robotically assisted insertion of acetabular or S2-alar-iliac [57–59] screws, although some literature sources [56, 60] have already mentioned and discussed the possibilities and potential benefits for these areas of application. As one example of many, it is important to mention the S2-alar-iliac screw. These screws can be perfectly inserted through the often narrow anatomical corridor available even minimally invasive. From our point of view, this is often difficult to realise with conventional techniques and only at increased risk. The insertion of screws in the pelvic area is in general very challenging due to the complex anatomical structures [55]. Navigation in combination with robotics can be an excellent surgical treatment option for the pelvic area by increasing accuracy and thereby increasing safety for the patient.

Three studies [57–59] were mentioned above, which evaluated their accuracy by measuring the cortical breach. Accuracy rates of the studies, ranged from 93.8% [57] to 95.7% [58], respectively only accurate screw position [59] was achieved. These studies were probably faced with the same challenge as we were, to quantify the accuracy without a standardised classification like the Gertzbein and Robbins classification [20, 24] on the spine.

In our view, therefore the accuracy for non-spinal screws does not have the same validity as the accuracy of spinal screws. The latter could be graded according to a more standardised and recognised classification, the Gertzbein and Robbins classification [20, 24].

#### Overall accuracy of screw placement

The evidence from the literature shows that it is entirely plausible that navigated and robotically assisted procedures can increase accuracy compared to other conventional techniques and purely navigated surgery [13, 30, 45, 51, 61, 62]. This may be primarily due to the ability of the 3D-Navigation Hybrid OR to reduce surgeon fatigue and minimized tremor. Both of which are important factors that have already been recognized in the literature as surmountable by robotics [63].

### Key elements for achieving a good surgical result in a Hybrid OR

There are multiple factors in a Hybrid OR which can improve the surgical outcome. Even though many of these factors are difficult to quantify from a scientific point of view, we would like to contribute to the discussion of the main factors influencing a good surgical outcome in a hybrid operation theatre.

In our opinion, the crucial factors sorted by impact might be:

1. **Cone beam CT**: CBCT imaging is at the heart of the hybrid operating theatre. High quality imaging is crucial to visualize where the lesion and surrounding structures are located. This is the only way to work precisely and achieve high levels of accuracy. Without high resolution imaging, there is neither a good basis for image fusion and registration nor a good basis for the additional positive effect of the robotic arm on accuracy.
2. **Registration/Fusion**: Simple, fast and accurate fusion and registration is also essential for good operative performance. In addition to the entirely technical system aspects, it is up to the surgeon to inspect the fusion and to check it at the end of the fusion process. Independent of the precision and conscientiousness of the surgeon’s work, the desired outcome will not be achieved if this step is not carried out carefully.
3. **Preoperative planning**: In our view, planning is a crucial step preoperatively. It saves time and capacities to do the planning usually one day before the operation in order to get familiar with the individual anatomy of the patient. Furthermore, precise planning without time pressure during the operation will result in high accuracy. Bad screw planning will result in poor screw positions.
4. **Robotic Arm**: The use of the robotic arm is a helpful addition to the pure freehand navigation. The assistive robotic arm significantly helps to save and secure the direction of the trajectory for the intended borehole.
5. **The elimination of the surgeon’s individual confounding factors** by the robotic arm has a positive effect. However, it should be noted that the arm works on the basis of the points mentioned above. As soon as the robotic arm is used, all the points mentioned above are brought together. It is therefore of the utmost importance to perform the mentioned factors in the best possible way.
6. **Technical support in the OR**: As we see it, the presence of a specially trained technical employee from the manufacturer has a number of benefits for an operation in a Hybrid OR. Particularly worth mentioning here is the assistance during the registration and fusion process, the first level support as well as the preparation and follow-up of the technical equipment in the OR. Thanks to his expertise, this employee can thus contribute to an optimal workflow in a hybrid operating theatre.

As the points are interlinked, it is difficult to emphasise isolated issues. Ultimately, good execution of each step is enormously important for the overall surgical outcome in a Hybrid OR.

#### Limitations

This study has several limitations. Surgeries of varying severity and duration were carried out over the entire study period. Therefore, no periods can be meaningfully subdivided, as there is no comparability. For this reason, no profound statement can be made about a possible learning curve regarding the development from the durations of the surgeries performed. Even though our aim was to record the entire occupancy time of the operating theatre, this was not possible in our study. Certainly, existing studies indicate a decrease in the duration of occupancy of the operating theatre [61]. Further, it is also difficult to make a high-quality statement about the hospital length of stay. Although previous studies indicated shorter length of hospital stay [33, 34, 64], as mentioned above, these studies are often limited to a small area of intervention. Our cohort of patients was a mixture of various ages, indications, underlying diseases and range of surgical treatments. This might have an additional influence on the length of hospital stay making it difficult to compare as an outcome parameter. Furthermore, it must be mentioned here that non-spinal screws could not be evaluated according to a standardized and validated classification. As a result, the accuracy of these screws cannot be considered comparable to the accuracy of spinal screws.

Finally, it is important for us to emphasize that further limitations lay in the study design, as this was a prospective observational study with retrospective analysis performed in a single centre with no control group.

## Conclusion

By extending the scope of application, this study showed that interventions in a fully equipped 3D-Navigation Hybrid OR can be successfully performed not only on the spine, but also on the pelvis and the extremities. In trauma, orthopaedics and spine surgery, navigation and robotics can be used to perform operations with a high degree of precision, increased safety, reduced radiation exposure for the OR-team and a very low complication rate.

## Data Availability

The data and material are not available due to the authors institute’s data protection policy.

## Abbreviations

AIR: Automatic Image Registration
CBCT: Cone beam computed tomography
CDC: Centers for Disease Control and Prevention
CI95%: 95% confidence interval
CT: Computed tomography
CTDI: Computed tomography dose index
Cx: Cervical spine level
DAP: Dose area product
DICOM: Digital imaging and communications in medicine
E.g.: Exempli gratia
et al.: et alia
Sx: Sacral spine level
GR: Gertzbein and Robbins
Gy: Gray
Hybrid OR: Hybrid operating room
i.e.: id est
ISS: Injury severity score
Lx: Mlumbar spine level
MIS: Minimally invasive surgery
mSv: Millisievert
OR: Operating room
PACS: Picture archiving and communication system
RDSR: Radiation dose structured report
SD: Standard deviation
SI-screws: Sacroiliac screws
TLIF: Transforaminal lumbar interbody fusion
Tx: Thoracic spine level
2D: Two-dimensional
3D: Three dimensional
